# Application of Deep Learning in Civil Engineering Management

**DOI:** 10.1155/2022/5372384

**Published:** 2022-01-28

**Authors:** Hongbin Zhao

**Affiliations:** China State Construction Engineering Corporation Limited, Beijing 100037, China

## Abstract

Construction safety issues are of great significance in civil engineering management. In this paper, the entry point is the recognition of workers wearing helmets during the construction process, and the recognition performance is improved by combining deep learning and traditional classifiers to achieve intelligent recognition of construction safety clothing. In the specific process, the deep residual networks (ResNet) and sparse representation-based classification (SRC) are used as basic classifiers to classify samples with unknown categories. The results of the two decisions are fused and the reliability of the fused decision is determined. Afterwards, the reliable test samples are added to the original training samples to update the classifier, so as to obtain more reliable recognition results. The proposed method is tested and verified with actual measured data. The experimental results show the effectiveness and robustness of the proposed method.

## 1. Introduction

Safety in production is an eternal theme in civil engineering management. The traditional construction procedure and management have a series of problems such as low safety management level, small management scope, large subjective interference, and poor timelines. In addition, it mainly relies on the subjective monitoring of safety managers and unable to monitor the whole process, so that accidents happen frequently [1–3]. At the same time, in the past project management process, safety officers mainly used experience analysis to reduce the occurrence of accidents, but it is impossible to completely accurately prevent various safety hazards in the entire life cycle of the project during the design and planning stage [4–6]. Therefore, an automated safety detection method for workers wearing helmets helps to improve the safety management level of the construction site. In recent years, the popularity of cameras in construction sites and the efficient application of deep learning in speech recognition, image recognition, and natural language processing have provided a new perspective for the safety management of construction sites. Using intelligent methods to replace traditional manual monitoring is conducive to real-time on-site monitoring, which not only saves labor costs but also improves on-site safety [7–10].

Aiming at the problem of construction safety, this paper puts forward a method for identifying workers wearing helmets based on deep learning algorithms. The core of the method in this paper is to use test samples whose categories can be reliably classified and confirmed in the classification process to optimize the original classifiers, so as to obtain stronger classification performance. Specifically, the initial residual network (ResNet) [11–14] is first designed as the dominant classifier for helmet target recognition. In addition, this paper chooses the sparse representation-based classification (SRC) as the auxiliary classifier and confirms the classification of the samples to be recognized together with ResNet. The original training samples are used to train ResNet, and at the same time, they are used to construct a global dictionary of SRC. For a certain test sample to be identified, it is classified by ResNet and SRC respectively to obtain the corresponding decision vectors, and then the final decision variable is obtained through weighted fusion. The design criteria determine the category credibility of the current test sample. The test sample is added into the original training samples when the conditions are met. So, the augmented training set can optimize the ResNet and expand the scale of the SRC global dictionary [15–18]. With the continuous increase of test samples with confirmed categories, the classification performance of ResNet and SRC can be continuously enhanced, and the recognition results obtained by fusion are more reliable. The main innovations of the method in this paper are as follows: (1) an update mechanism is introduced under the decision fusion framework of ResNet and SRC classifiers. Classifier decision fusion is a common method to improve decision accuracy. In the traditional method, the classified test samples are not fully used, and the online update of the classifier is lacking. In the problem of target recognition in helmet images, the number of limited training samples is very limited. This paper confirms the test samples and updates the classifier, which can effectively improve the classification ability of the classifier. (2) A criterion for decision-making reliability is proposed and used for the screening of test samples. Although the accuracy of the result of fusion decision is improved, there is still the probability of misclassification. The introduction of test samples with the wrong decisions will result in a decrease in the performance of updating the classifier. Based on the fusion of probabilistic decision variables, this paper defines decision reliability indicators to select test samples whose decision reliability is higher than the preset threshold to update the classifier, so as to ensure the effectiveness of the update of the classifier [19–25]. Experiments are conducted on the measured dataset; the results show that the proposed method has performance advantages compared to a single classifier and traditional classifier decision fusion methods.

## 2. Principle of Classifiers

### 2.1. ResNet

Deep convolutional neural networks (CNN) have brought a series of breakthroughs to computer vision. However, as the depth of the model increases, the performance of the model also degrades. He et al. conducted research on the degradation of deep models and proposed ResNet, which solved the problem of performance degradation of CNN under conditions of increased depth, and promoted the performance of computer vision tasks, especially the image recognition performance.

**Figure 1:**
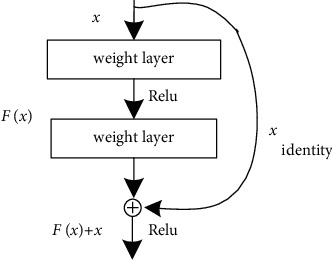
A building block in ResNet.

The idea of residual learning is to assume that the deep layer of the model is an identity mapping, and the problem that the model needs to solve is to learn the identity mapping function. However, *H*(*x*)=*x* is more difficult to directly fit the identity function and *F*(*x*)=*H*(*x*)+*x* and *H*(*x*) are easier to fit the residual function. Therefore, He et al. proposed a residual unit, which uses a cross-layer connection method to replace the original mapping and convert it into a learned residual function. Let *F*(*x*)=*H*(*x*) − *x* and *F*(*x*)=0; an identity mapping *H*(*x*)=*x* is constituted. The structure of the basic residual unit is shown in Figure 1. The network does not generate additional parameters, nor does it increase computational complexity.

The input *x* and output *y* of the residual unit can have different dimensions. When the dimensions are the same, equation (1) can be directly executed. When the dimensions are different, equation (2) can be executed to match *x* with *W*_*s*_, where *F*(*x*, {*W*_*i*_}) is the residual function to be fitted:

(1) $$\begin{array}{c} y=F\left(x,\left\{W_{i}\right\}+x\right), \end{array}$$

(2) $$\begin{array}{c} y=F\left(x,\left\{W_{i}\right\}+W_{s}x\right). \end{array}$$

According to existing literature reports, ResNet has strong processing capabilities for image recognition problems. For this reason, this paper adopts ResNet as one of the basic classifiers for the identification of construction workers' helmet wearing problems.

### 2.2. SRC

SRC uses sparse representation for pattern recognition problems, which characterizes unknown inputs through training samples of known types and then determines the types of test samples based on different types of reconstruction errors [13–16]. Suppose *D*=[*D*^1^, *D*^2^,…, *D*^*C*^] ∈ *R*^*d*×*N*^ is a global dictionary, where *D*^*i*^ ∈ *R*^*d*×*N*_*i*_^(*i*=1,2,…, *C*) represents the *N*_*i*_ training samples from the *i*th class. For the test sample *y*, the sparse representation process is as follows:

(3) $$\begin{array}{c} \hat{x}=\underset{x}{\arg\min}{\left\|x\right\|}_{0},\\ \text{s.t.}{\left\|y-Dx\right\|}_{2}^{2}\leq \varepsilon . \end{array}$$

In the equation, *x* represents the sparse coefficient vector. At this stage, algorithms commonly used to solve sparse representation problems include the *ℓ*_1_ norm optimization and orthogonal matching pursuit algorithm (OMP) [13–16]. According to the solution result of equation (3), i.e., $\hat{x}$, the reconstruction error for the test sample is calculated by category, and the target label of the test sample is finally determined as follows:

(4) $$\begin{array}{c} r\left(i\right)={\left\|y-D_{i}x_{i}\right\|}_{2}^{2},\;\left(i=1,2,\ldots ,C\right),\\ \text{identity}\left(y\right)=\underset{i}{\arg\min}\left(r\left(i\right)\right), \end{array}$$

where *x*_*i*_ is the coefficient vector corresponding to the *i*th class; *r*(*i*) is the corresponding reconstruction error.

Compared with ResNet, the classification mechanism of SRC is relatively less dependent on the number of test samples. At the same time, existing research results show that SRC has certain adaptability to complex situations such as noise interference and occlusion. Therefore, ResNet and SRC have a certain degree of complementarity in classification decision-making. So, the fusion of the decision-making results of the two classifiers is conducive to obtaining more reliable recognition results.

## 3. Classifier Update and Recognition Method

### 3.1. Decision Criteria and Classifier Update

For ResNet and SRC classification results, this paper uses a weighted fusion method to get the final decision variables. The decision variable output by ResNet is the posterior probability vector [*P*_1_, *P*_2_,…, *P*_*C*_]. For SRC, the output result is the reconstruction error vector [*r*_1_, *r*_2_,…, *r*_*C*_]. First, the SRC decision variable is converted into a probability vector according to the following equation:

(5) $$\begin{array}{c} P_{i}^{S}=1-\frac{r_{i}}{\sum_{j=1}^{C}r_{j}}. \end{array}$$

On this basis, the classic weighted (equal weight) algorithm [24, 25] is used to fuse the posterior probability vector of ResNet and the transformed posterior probability vector of SRC, as shown below:

(6) $$\begin{array}{c} P_{i}^{F}=0.5*P_{i}+0.5*P_{i}^{S},\;i=1,2,\ldots ,C. \end{array}$$

According to the final decision variables [*P*_1_^*F*^, *P*_2_^*F*^,…, *P*_*C*_^*F*^], this paper defines decision reliability as follows:

(7) $$\begin{array}{c} R=\min\left(\frac{P_{K}^{F}}{\max\left(P_{i}^{F}\right)}\right),\;\left(i\neq K\right), \end{array}$$

where *P*_*K*_^*F*^ is the maximum probability value, and *R* ≥ 1.

Correspondingly, the larger the value *R*, the more reliable is the classification result. An appropriate decision threshold can be set. When the decision reliability is higher than the threshold, the current decision is considered to be reliable, and the corresponding test sample is added to the original training samples to update the ResNet and SRC classifiers. Otherwise, the training set is not updated.

### 3.2. Identification Process

In this paper, the training set is dynamically updated by analyzing the reliability of decision-making to obtain more reliable SRC and ResNet classifiers. The key steps of the specific implementation are summarized as follows:

  Step 1: the original training samples are used to train the ResNet and construct the SRC global dictionary at the same time;
  Step 2: for test samples of unknown categories, ResNet and SRC are used to classify them, respectively. Their categories are determined and the reliability of the fused decision is calculated;
  Step 3: if the decision-making reliability of the current test sample is higher than the preset threshold, it is added to the original training samples to dynamically update the ResNet and SRC global dictionary;
  Step 4: repeat steps 2, 3, and 4 for all test samples until all samples are classified.

With the increasing number of test samples for category confirmation, the updated ResNet and SRC classification capabilities have also been continuously enhanced.

## 4. Experiment and Analysis

### 4.1. Experimental Data and Conditions

This paper uses surveillance video data from a mineral enterprise over a period of time and converts the video into 5000 pictures. These images are preprocessed appropriately to increase the amount of data and the generalization ability of the model. The collected images are divided into 3 categories. The first category is the mine background (denoted as “Background”) with a total of 1200 pictures. The second category is the mine worker wearing a safety helmet (denoted as “Safe”) with a total of 2500 pictures. The third category is a total of 1,300 pictures of mine workers (denoted as “Unsafe”) who do not wear safety helmets. In the specific experiment, 600 “Background” pictures, 1300 “Safe” pictures, and 700 “Unsafe” pictures are used as training samples, and the remaining samples are used for testing.

Subsequent experiments will be carried out under three types of conditions. The first category is called basic testing, which mainly tests and validates the proposed method on the original training and test sets. This condition is relatively simple, and the basic performance of the method is mainly investigated. The second category is noise interference. The noise sample set is constructed by adding different degrees of Gaussian white noise simulation to the original test samples to test the adaptability of the proposed method to noise interference. The third type is partial occlusion. Due to problems such as occlusion and viewing angle, some of the collected images may be partially missing. This paper simulates the situation of partial occlusion through image processing and constructs test sets at different occlusion levels, and then the experiment tests the adaptability of the proposed method to occlusion conditions.

The experiments also set up contrast methods, mainly including “SRC,” “ResNet,” and “parallel fusion.” The first two comparison methods mainly use a single classifier for recognition. The third method also uses ResNet and SRC in this paper as the basic classifiers, but only simple parallel fusion is performed in the fusion stage, which lacks the process of classifier update.

### 4.2. Experimental Results and Discussion

#### 4.2.1. Basic Test

The method in this paper is used to classify the original samples of the three types of images, and the statistical results shown in Table 1 are obtained. The recognition rates of “Background,” “Safe,” and “Unsafe” images are 97.67%, 96.83%, and 98.33%, respectively, and the average recognition rate is calculated as 97.42%. This result shows the effectiveness of this method for image recognition of helmet wearing. The three types of comparison methods are tested under the same conditions, and the average recognition rates of all methods are shown in Table 2. The comparison shows that the recognition performance of the proposed method under basic test is better than those of the comparison methods, which verifies its stronger effectiveness. Compared with the method that uses SRC or ResNet alone, this paper confirms the test samples and supplements the training set and organically combines the classification results of the two to significantly improve the final classification accuracy. Compared with the traditional simple parallel fusion method, the method in this paper dynamically updates the two classifiers while fusing ResNet and SRC, and the enhancement in the final recognition performance is also very obvious.

#### 4.2.2. Noise Interference

**Figure 2:**
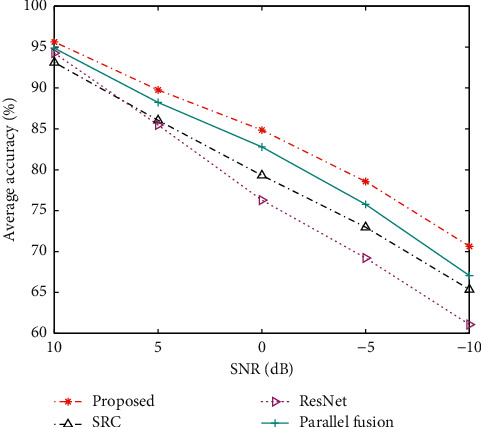
Comparison of performance under noise corruption.

In order to test the performance of the proposed method under noise interference conditions, based on the original training and test sets, different degrees of noises are added to the original test samples to construct test sets with different signal-to-noise ratios (SNR). Various methods are tested at different SNRs, and the results are shown in Figure 2. It can be seen that the method in this paper maintains the highest recognition rate at each noise level, showing its better noise robustness. Since the training samples of the ResNet are all from high SNRs, its classification accuracy for noise interference, especially for test samples under a low SNR, is significantly reduced. Compared with ResNet, the SRC method is more adaptable to low-SNR samples, mainly due to the robustness of sparse representation to noise interference. The proposed method maintains the inherent noise robustness of SRC through the combination of ResNet and SRC and also improves the coverage of noise interference situations by dynamically updating training samples. So, the final fusion decision is more targeted for noise samples.

#### 4.2.3. Partial Occlusion

In the actual enterprise construction process, the video surveillance may have insufficient field of view, or the surrounding occlusion may cause partial occlusion of the collected images. Therefore, the performance of the recognition method under partial occlusion conditions is also critical. Based on the original test sample, this experiment simulates the partially occluded test sample by removing part of the image. The occlusion level is evaluated by the proportion of missing images. Afterwards, this experiment constructs four test sample sets with different occlusion ratios of 10%, 20%, 30%, and 40% and tests various methods. The results are shown in Table 3. It can be seen that the method in this paper maintains the highest recognition performance under various occlusion conditions, showing its robustness. Similar to the case of noise interference, the method in this paper uses the organic fusion of two classifiers to play their complementary role, which is conducive to improving the overall recognition performance.

## 5. Conclusion

For the construction safety issues in civil engineering management, this paper proposes a method of target recognition for helmet wearing images based on updating the classifier. The available training samples are continuously updated by confirming the types of test samples, thereby improving the classification performance. Using ResNet and SRC as the basic classifiers, the independent classification performance is improved on the basis of updating the training samples. At the same time, the two classifiers are fused at the decision-making level to obtain a more reliable recognition result. Experiments are carried out based on the measured dataset, and the classification performance of the proposed method is tested under basic conditions, noise interference, and partial occlusion scenarios, while compared with other methods. The experimental results show that the recognition performance of the method in this paper is better than the comparison method under all conditions, verifying its effectiveness and robustness. In the follow-up research, this paper will further organically combine deep learning detection algorithms and identification methods to form an end-to-end closed-loop security detection and identification method in order to further enhance the company's security management capabilities.

## Figures and Tables

**Table 1:** Recognition results of the proposed method under basic test.

Actual label	Predicted label			Accuracy (%)
	Background	Safe	Unsafe	
Background	586	6	8	97.67
Safe	20	1162	18	96.83
Unsafe	3	7	590	98.33

**Table 2:** Comparison of performance under basic test.

Method	Average accuracy (%)
Proposed	97.42
SRC	95.78
ResNet	96.54
Parallel fusion	96.89

**Table 3:** Average accuracy of different methods under partial occlusion (%).

Method	Occlusion level (%)			
	10	20	30	40
Proposed	95.88	90.05	82.45	70.13
SRC	93.24	87.53	78.23	67.89
ResNet	94.27	87.02	77.54	65.84
Parallel fusion	95.02	88.56	80.42	68.19

## Data Availability

The dataset can be accessed upon request.
